# The prefusion structure of herpes simplex virus glycoprotein B

**DOI:** 10.1126/sciadv.abc1726

**Published:** 2020-09-25

**Authors:** B. Vollmer, V. Pražák, D. Vasishtan, E. E. Jefferys, A. Hernandez-Duran, M. Vallbracht, B. G. Klupp, T. C. Mettenleiter, M. Backovic, F. A. Rey, M. Topf, K. Grünewald

**Affiliations:** 1 Oxford Particle Imaging Centre, Department of Structural Biology, Wellcome Centre Human Genetics, University of Oxford, Oxford, UK.; 2 Centre for Structural Systems Biology, Heinrich-Pette-Institut, Leibniz-Institut für Experimentelle Virologie, Hamburg, Germany.; 3 Department of Biochemistry, University of Oxford, Oxford, UK.; 4 Institute of Structural and Molecular Biology, Birkbeck, London, UK.; 5 Friedrich-Loeffler-Institut, Federal Research Institute for Animal Health, Insel Riems, Germany.; 6 Institut Pasteur, Structural Virology Unit, Department of Virology, Paris, France.; 7 Department of Chemistry, MIN Faculty, Universität Hamburg, Hamburg, Germany.

## Abstract

Cell entry of enveloped viruses requires specialized viral proteins that mediate fusion with the host membrane by substantial structural rearrangements from a metastable pre- to a stable postfusion conformation. This metastability renders the herpes simplex virus 1 (HSV-1) fusion glycoprotein B (gB) highly unstable such that it readily converts into the postfusion form, thereby precluding structural elucidation of the pharmacologically relevant prefusion conformation. By identification of conserved sequence signatures and molecular dynamics simulations, we devised a mutation that stabilized this form. Functionally locking gB allowed the structural determination of its membrane-embedded prefusion conformation at sub-nanometer resolution and enabled the unambiguous fit of all ectodomains. The resulting pseudo-atomic model reveals a notable conservation of conformational domain rearrangements during fusion between HSV-1 gB and the vesicular stomatitis virus glycoprotein G, despite their very distant phylogeny. In combination with our comparative sequence-structure analysis, these findings suggest common fusogenic domain rearrangements in all class III viral fusion proteins.

## Teaser

Elusive, pharmacologically relevant conformation of the herpesvirus fusion protein is revealed by a multi-methodological approach.

## INTRODUCTION

Herpesviruses present a burden to the general public health as they comprise important human pathogens like herpes simplex virus (HSV-1), varicella-zoster virus (VZV), Epstein-Barr virus (EBV), and cytomegalovirus, the latter being one of the leading viral causes of birth defects in developed countries. Still, there is a lack of vaccines against most of these pathogens. For all enveloped viruses, specialized surface proteins catalyze the fusion of viral and cellular membranes for entry (*1*) and also represent a primary target for the immune system. There are three structural classes of viral membrane fusion proteins, but they all appear to function via a similar mechanism despite their radically different three-dimensional structures. Membrane merging is achieved via substantial structural rearrangements of these proteins from a metastable pre- to a stable postfusion conformation. The prefusion form presents an attractive target for drug and vaccine development as demonstrated for the class I fusion protein F of respiratory syncytial virus (RSV), for which the most potent neutralizing antibodies target this conformation only (*2*). Identifying ways to stabilize the prefusion conformation of RSV F has been a major advance in the development of surface epitope-focused, next-generation vaccines (*3*, *4*). Herpesvirus glycoprotein B (gB) is a structurally conserved, class III membrane fusion protein, composed of α- and β-secondary structure elements, that combines structural features characteristic of class I and II fusion proteins (*5*). Although the gB postfusion conformation was determined more than 10 years ago (*6*), only recently first descriptions of the prefusion form were reported (*7*, *8*). Attempts to determine high-resolution structures by crystallization or single-particle analysis electron cryo microscopy (cryo-EM) failed because of its metastability, causing it to readily adopt the postfusion conformation when using purification methods involving membrane anchor or lipid removal (*9*). Even on native membranes, mixtures of full-length gB trimers present in either pre- or postfusion conformations are found (*7*, *10*). Stabilization of gB in the prefusion conformation allowing determination of its structure and comprehensive domain model will greatly assist structure-based drug design and antibody generation. Here, by using a molecular dynamics (MD) simulations and sequence-structure analysis guided mutational approach, we successfully trapped the prefusion conformation of gB and determined its structure to sub-nanometer resolution by electron cryo tomography (cryo-ET) and sub-volume averaging (SVA). The quality of the cryo-ET map allowed an unambiguous description of the domain arrangements, highlighting the substantial structural changes required to drive the membrane fusion reaction and a notable resemblance to the conformational changes described for vesicular stomatitis virus glycoprotein G (VSV-G).

## RESULTS

### A conserved hinge in DIII of gB

**Fig. 1:**
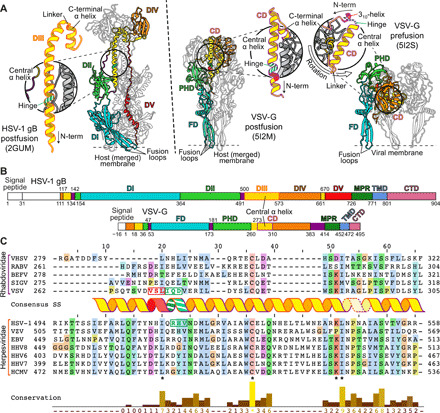
Determination of a conserved hinge in DIII of gB. (**A**) Trimeric ectodomain x-ray structures of gB in postfusion (*6*) and VSV-G in postfusion (*11*) and prefusion (*12*) conformation. Corresponding domains are depicted using the same color scheme for a single protomer. The central helices of each of the protomers are magnified and displayed in insets. The hinge regions in gB RHV^515–517^ and VSV-G IQD^272–274^ are marked in striped green and the 3_10_-helix VSL^269–271^ in red. Location of fusion loops is marked for one protomer. Domains are named according to (*6*) for gB and (*16*) for VSV-G. CD, central domain; PHD, pleckstrin homology domain; FD, fusion domain. (**B**) Domain architecture of gB and VSV-G. Numbers indicate amino acid positions of the domain boundaries. N-terminal signal peptides and the unstructured N-terminal domain of gB are shown in white, and flexible linker regions are shown in purple. CTD, C-terminal domain. (**C**) Sequence alignment of central helices of glycoprotein G [domain II (DII)] of *Rhabdoviridae* and glycoprotein B (DIII) of human Herpesviridae. The consensus secondary structure of the central postfusion coiled-coil helix of VSV (*11*) and HSV-1 (*6*) is shown in between the alignment of the two virus families. The color outlining the helix indicates the length of the helix of VSV-G (purple) and HSV-1 gB (orange), respectively, while the dashed outline marks the linker region to the C-terminally attached short helix. The residues forming the N-terminal 3_10_-helix of the prefusion VSV-G structure are shown in red letters, and the hinge region residues of the same structure as well as the putative hinge in HSV-1 gB are shown in green letters. Residue background coloring is based on Clustal Omega (*39*), and conservation was calculated using www.compbio.dundee.ac.uk/aacon/ (*40*). Three highly conserved, sequence signatures are marked by asterisks on the bottom of the alignment. VHSV: viral hemorrhagic septicaemia virus; RABV: rabies virus; BEFV: bovine ephemeral fever virus; SIGV: *Drosophila melanogaster* sigma virus; VZV: varicella zoster virus; EBV: Epstein-Barr virus; HHV8: Kaposi’s sarcoma-associated herpesvirus; HHV6: human herpesvirus 6; HHV7: human herpesvirus 7; HCMV: human cytomegalovirus.

gB of the double-stranded DNA (dsDNA) herpesvirus HSV-1 and glycoprotein G of the single-stranded RNA (ssRNA) rhabdovirus VSV are the archetypic members of class III fusion proteins (*6*, *11*). Like all known members of this class, these are single membrane–spanning proteins that form trimers. Despite belonging to evolutionary seemingly unrelated virus families, the arrangement and structure of their individual domains are conserved (Fig. 1, A and B), but high-resolution structures for the prefusion form are only available for two rhabdovirus glycoproteins (*12*, *13*). In the postfusion conformation (Fig. 1A), domain I (DI) of gB is mainly formed by β sheets, exposing the fusion loops that insert into the target membrane (*6*, *14*, *15*). Moving away from the membrane, the polypeptide chain then folds into domain II (DII), which consists of α helices and β sheets, continuing into domain III (DIII) that features a prominent, centrally located α helix and forms a long trimeric parallel coiled-coil at the center of the gB trimer. At the top of the protein is the globular domain IV (DIV), followed by an extended segment, domain V (DV). The latter runs along the entire trimer down to the membrane-proximal region (MPR) and membrane anchor located at the same end as the fusion loops. In VSV-G, a similar arrangement is formed by conserved, but shorter, domains with gB DV being substituted in VSV-G by a mostly disordered stem region leading straight into the MPR (Fig. 1B).

**Fig. 2:**
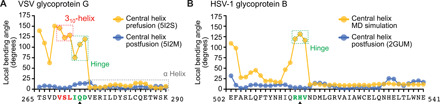
MD simulation of DIII in solution. (**A**) Local bending angles of VSV-G DII in prefusion (yellow) (*12*) and postfusion (blue) (*11*) conformation. Protein Data Bank (PDB) numbers are given in parentheses. Amino acids are given in one-letter code. The 3_10_-helix and hinge region are highlighted by dashed boxes in red and green, respectively. The α-helical part is highlighted by dashed box in gray. The position permitting a helix breaking mutation is marked with a black arrowhead. (**B**) Local bending angles of HSV-1 gB DIII in MD simulation (yellow) and postfusion (*6*) conformation (blue). The predicted hinge region in the MD simulation is marked in green. The position permitting a helix breaking mutation is marked with a black arrowhead.

Comparative analyses of the pre- and postfusion structures of VSV-G (*11*, *12*) revealed that its conformational transition is largely achieved by interdomain rotations, occurring mainly between the fusion domain (FD) containing the fusion loops and the pleckstrin homology domain (PHD), and a notable refolding of the region N-terminal to the central domain (CD), corresponding to the C terminus of DIII and DIV in gB [Fig. 1, A (insets) and B] (*5*, *16*). In the prefusion conformation of VSV-G, the central helix consists of a 3_10_-helix (amino acids 269 to 271) oriented perpendicular to the α-helical region immediately downstream (amino acids 275 to 293) (*12*), the two being connected via a short hinge (amino acids 272 to 274; Figs. 1, A and C, and 2A). In the postfusion form, the entire region (amino acids 263 to 294) refolds into a single, long α helix that subsequently forms the central coiled-coil of the trimer (Figs. 1A and 2A).

Even though the domain containing this central helix shows a high diversity in sequence and length between class III fusion proteins, we identified three sequence signatures occurring in fusion proteins of the families Rhabdoviridae and Herpesviridae (Fig. 1C): (i) an L or I residue located at the N terminus of the central helix; (ii) a conserved cysteine residue forming a disulfide bond; and (iii) a KΦ (Φ indicating a hydrophobic residue) combination toward the C terminus of the α-helical part, which is followed by a linker and a short α helix, kinked away from the main helix (Fig. 1A). Notably, in VSV-G, the highly conserved leucine of the first signature is located at the edge of the 3_10_-helix, the segment that undergoes refolding during the pre- to postfusion transition. We therefore focused on the corresponding region in DIII of gB.

### MD simulation of DIII in solution

To test whether the N-terminal part of the central helix is also important for the pre- to postfusion transition in HSV-1 gB and to pinpoint specific residues involved, we performed MD simulations using a “reverse engineering” in silico approach, starting from the long central helix (DIII) of the gB postfusion form (Fig. 1A). In two independent 100-ns MD simulations, a hinge readily formed after 10 and 9 ns, respectively, and remained stable for the rest of the simulations (movie S1). In plots of the local bending angle, this hinge can be identified as peak in the simulated helix similar to the prefusion conformation of VSV-G (Fig. 2A), suggesting that the hinge region in gB is located within residues 515 to 517 (Fig. 2B).

### Locking the gB prefusion conformation

**Fig. 3:**
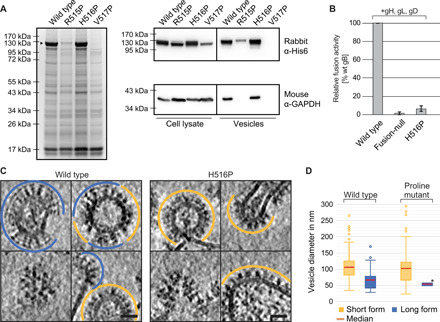
Locking and functionally arresting the gB prefusion conformation. (**A**) SDS–polyacrylamide gel electrophoresis (PAGE) and Coomassie stain of extracellular vesicle purifications, obtained from cells transfected with the HSV-1 gB wild type (wt) and single-point mutants, and Western blot analysis of the cell lysate and extracellular vesicles. Mature gB protein is marked by a black arrowhead. Used antibodies are indicated on the right. GAPDH: glyceraldehyde-3-phosphate dehydrogenase. (**B**) Fusion activity of gB wt, a fusion-null construct, or a single-point mutant in combination with gH/gL and gD in a cell-cell fusion assay. Activities are normalized to wt fusion activity level. (**C**) Cryo-ET slices of purified extracellular vesicles formed by WT and gB H516P. The long, postfusion form of gB is indicated by blue lines, and the short form is indicated by orange lines. Lower left images show the top views of gB trimers. Scale bars: 25 nm. (**D**) Size distribution of vesicles found with short and long gB form on vesicles formed by wt gB (*n* = 183) and gB H516P (*n* = 114). Boxes indicate range from first to third quartile with whiskers showing ±1.5 interquartile range and outliers marked as points. *no range given, as only two vesicles were found displaying the long form of gB H516P. The red line marks the median diameter.

On the basis of this finding, we tried to stabilize the DIII central helix hinge and, thus, the prefusion form of gB. Previous attempts to do so, by mutations or short linker insertions in DIII (*17*, *18*), have failed and only yielded the postfusion conformation. Here, we combined an approach successfully applied to stabilize class I fusion proteins in their prefusion form (*4*, *19*–*21*) with our method to display full-length, membrane-anchored gB on extracellular vesicles (*22*). For this, we introduced helix-breaking point mutations to proline into the MD-predicted helix hinge at residues 515 to 517 of gB (Figs. 2B and 3A). This proline scanning resulted in wild type (WT)–like expression of full-length mutant gB only with a proline at position 516 (Fig. 3A). Mutations in adjacent positions resulted in low or no expression of mature protein and absence of vesicles (Fig. 3A). Notably, mutations in the corresponding VSV-G hinge region (amino acids 272 to 275) between the 3_10_ and the downstream α helix were also tolerated in one position (Q273) only (fig. S1, A and B). The amount and ratio of fully to partly glycosylated gB on extracellular vesicles, detectable as a single or double band, respectively (*23*), were not altered by the H516P mutation, suggesting that expression and posttranslational modifications were not affected (Fig. 3A).

### Locked gB is functionally arrested

We then tested the fusion activity of gB H516P in a cell-cell fusion assay (*24*) in the presence of gD, gH, and gL. All four glycoproteins are essential and sufficient for HSV-1 membrane fusion and entry (*25*, *26*). Compared to wt gB, the cell-cell fusion activity of gB H516P was reduced to 6.5% (SD: 3.2%) (Fig. 3B), only slightly above the background level [1.6% (SD: 1.4%)] of a fusion-null construct (*27*, *28*). This fusion inhibition is consistent with a block in the pre- to postfusion state transition, functionally locking the protein.

### Displaying locked gB on native membranes

As we reported earlier, wt gB is present in two major forms on extracellular vesicles (Fig. 3C) (*7*): an extended one (~16 nm in length), corresponding to the postfusion conformation and mostly found on small vesicles ($\tilde{x}$ Ø = 59 nm), and a compact one (~12 nm), presumably corresponding to the prefusion state, found on larger vesicles ($\tilde{x}$ Ø = 98 nm) (Fig. 3, C and D). For wt gB, about 30% of vesicles predominantly presented the extended form, while 70% predominantly presented the compact form (*n* = 183), similar to previous observations (*7*).

For gB H516P, total vesicle production was similar to wt gB (Fig. 3A), but the ratio was drastically shifted to 98.2% (*n* = 112) of vesicles displaying solely the compact form and only 1.8% displaying the extended form (*n* = 2), with comparable size distributions as the wt vesicles ($\tilde{x}$ Ø = 53 nm for extended form; $\tilde{x}$ Ø = 90 nm for compact form) (Fig. 3D). Thus, introducing the H516P mutation apparently inhibited the transition of gB from the compact to the extended form. The small number of vesicles displaying the extended form of gB H516P parallels the remaining residual fusion activity, which is marginally above background levels (Fig. 3B). Notably, a gB H516P ectodomain construct (amino acids 31 to 730) lacking the MPR and transmembrane anchor crystallized in the postfusion conformation (fig. S2) corroborating the intrinsic propensity of gB ectodomain to easily adopt its postfusion form and the importance of the membrane anchor in maintaining the prefusion conformation.

### Structure of the locked gB

**Fig. 4:**
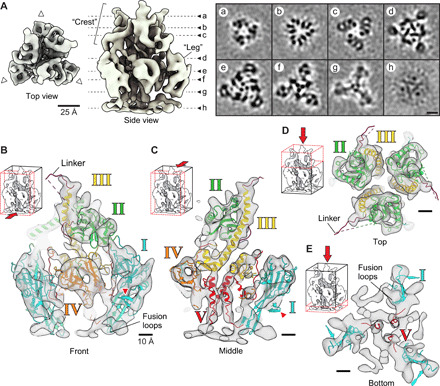
Structure of the locked gB protein. (**A**) Top and side view of the SVA structure, acquired by cryo-ET of gB H516P. Dashed lines mark the positions of the alphabetically marked cross sections on the right (a to h). White arrowheads point to the neighboring gB trimers. (**B**) Front slice of the fitted ectodomain structure showing the linker region connecting DII and DIII. Red arrowheads mark the β hairpin in DI protruding outside of the SVA map. Small insets at the top left indicate the direction of view and the section seen. Domains are marked and colored as in (*6*). (**C**) Middle slice showing the fit of DIII and DV in the central tubular densities. (**D**) Top view showing the crest region and fitted DII and DIII. (**E**) Bottom slice seen from above showing the fusion loops and central, tubular densities accommodating DV.

Next, purified vesicles displaying the H516P gB variant were structurally analyzed using cryo-ET and SVA. Earlier respective attempts were limited by the conformational flexibility (“breathing”) of the compact wt gB form. The relative homogeneity of the stabilized gB H516P protein on the vesicles greatly facilitated particle picking and averaging (Fig. 3, C and D). A total of 46,067 sub-volumes contributed to the final map (Fig. 4A) (Supplementary Materials) resolving the gB ectodomain at an overall resolution of 9 Å (fig. S3, A and B). This represents a substantial improvement in the level of structural detail compared to previous prefusion structures ranging in resolution from ~21 to 50 Å (*7*, *8*, *10*). The resolved ectodomain has clear C3 symmetry with overall dimensions of ~12 nm in height and ~10 nm in width. A three-pointed density (“crest”) crowns the membrane-connected trimer. Slices through the volume reveal a funnel-like arrangement of the crest (Fig. 4A, panels a to c). The lower part of the trimer is dominated by three major densities (“legs”) protruding laterally (Fig. 4A, top view, white arrowheads, and panels d to f), which frequently form interfaces with neighboring gB trimers (fig. S4) (*7*). These lateral interactions seem to be flexible, allowing the formation of pentameric, but also hexameric, arrangements on the membrane surface (fig. S4A). Most notably, particularly well-resolved rod-like densities (fig. S3A), with a diameter of ~8 Å and therefore most likely representing α helices, are located at the center of the trimer (Fig. 4A, panels e to g). A slice at the phospholipid headgroup level of the outer membrane leaflet reveals parts of the protein embedded in the membrane or connected to it (Fig. 4A, panel h).

### Domain architecture of the pre- and postfusion conformation

**Fig. 5:**
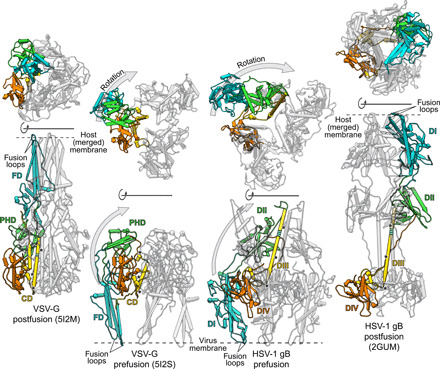
Comparison of the domain rearrangement between pre- and postfusion conformation in VSV-G and gB. The structures of VSV-G (*11*, *12*) and HSV-1 gB (*6*) are shown in side (lower row) and top (upper row) views in pipe representation with one protomer colored as in Fig. 1A. For clarity, DV of gB is omitted, and to increase visibility, one protomer is framed by a black silhouette. The prefusion conformation of each protein is depicted in the middle with the postfusion conformation on the sides. The pre- and postfusion conformation of each structure is aligned to the region including DIV (orange) and the C-terminal helix of DIII (yellow) in gB or the CD region in VSV-G, respectively. The one-sided gray arrows indicate the movement of DI (blue) and DII (green) in gB and the corresponding FD (blue) and PHD (green) in VSV-G in relation to the rest of the protein with the upward movement shown in the side views and the lateral movement shown in the top views. The hinge regions in DIII of gB and the CD in VSV-G are shown in striped green with the conserved sequence signatures marked by black asterisks.

**Fig. 6:**
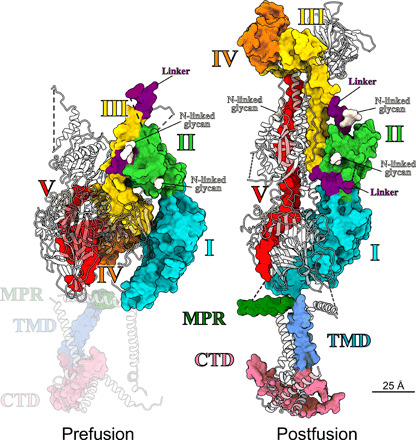
Domain architecture of the pre- and postfusion conformation. Structure of the rigid-body fit for the prefusion conformation in comparison to the full-length postfusion structure. Domains are marked and colored as in (*14*), also indicating representative positions of potential N-linked glycosylations. Two protomers per structure are shown as gray ribbons. For clarity, ribbons are half transparent. Because of the uncertainty of the fit, the MPR, TMD, and C-terminal domain (CTD) of the prefusion structure are shaded.

The resolution of our SVA map allowed the reliable placement of each individual domain of the gB ectodomain x-ray structure (*6*, *14*) by rigid-body fitting (*29*) and subsequent optimization by MD-based flexible fitting [Figs. 4 (B to E) and 6, and table S1] (*30*). The resulting model occupied essentially the complete density of the map (movie S2). The order of fitting the individual domains is depicted in movie S2 starting with DIV (orange), which forms the crown domain in the postfusion conformation (Fig. 1A). This domain showed the best fit being docked upside down, in relation to the membrane, into a fist-shaped density (table S1), located about 2 nm from the membrane (Fig. 4, B and C). This assignment placed DIII (yellow) in the central rod density. The short, kinked C-terminal helix of DIII (Fig. 1, A and C) fits well into the density that makes the connection between the central rod and DIV (Fig. 4C). At the top, the N terminus of DIII is connected to DII (green) via a flexible, ~3-nm-long linker region, predicted to form an unstructured poly-proline loop that was not resolved in the crystal structures (*6*, *14*). This connectivity led us to place DII in the crest density (Fig. 4, B and D). An alternative fit in the “leg” density would have required a longer linker length of at least 5 nm. DI (blue) was fitted in the leg density, connected to DII and leading to the membrane, localizing the fusion loops pointing toward the membrane (Fig. 4, B and C). The postfusion x-ray structure of gB shows a β hairpin in DI, perpendicular to the β hairpins exposing the fusion loops, for which no corresponding densities were resolved in our map (Fig. 4, B and C, red arrowheads). Notably, comparing the location of this β hairpin in gB of other herpesviruses showed some flexibility in relation to the rest of DI (*31*), indicating a possible conformational rotation that would place it inside our density map. This change could be due to subtle differences in DI between the pre- and postfusion conformations, which could explain why visually this fit appears not as good as those for DII to DV (Fig. 4, B and C). The central, tubular density leading toward the membrane most probably represents the previously unresolved DV (Fig. 4, C and E), which connects DIV with the MPR and transmembrane domain (TMD) helices (Fig. 4C). The close, ~1.5-nm-spaced arrangement of the three DV seen at the center of the map (Fig. 4A, panel g), compared to the ~3.6-nm distance in the postfusion structure (Figs. 1A and 5) (*14*), suggests an extensive reorientation of the connected amphipathic helices of the MPR discernible in our map (Fig. 4A, panel h, and fig. S5A). Because of the ~28–amino acid–long unresolved linker connecting DV to the MPR, two arrangements are possible, placing the TMD connections at different distances (~2.5 versus ~0.8 nm) from the molecular threefold symmetry axis (fig. S5A). An earlier study (*32*) suggested that the MPR amphipathic helices might function in shielding the fusion loops from the membrane in the prefusion conformation. On the basis of our sub-nanometer–resolution density map and rigid-body fitting, these two structural elements are too distant from each other to support this hypothesis. SVA further revealed, albeit at lower resolution, both membrane leaflets and a globular density underneath, connected via three distinct membrane contact points (fig. S5B). The latter density indicates the position of the cytoplasmic C-terminal domain (CTD) (*14*), which has been suggested to regulate gB fusogenicity (*33*). However, given the lower resolution of the map in this region, any density fitting of structural elements is still speculative (fig. S5B).

## DISCUSSION

The arrangement of secondary structure elements resulting from rigid fitting of the ectodomains I to IV accounts for the features resolved in the ectodomain EM map remarkably well (Fig. 4, B to D; table S1; and movie S2), although DI appears to undergo partial rearrangement during the fusogenic transition, which could place the β hairpin inside the resolved density. Fitting of DI into the density contacting the membrane is in contrast to our previous work where, using a 23-Å resolution map of gB (*7*), we placed DI at the top of the structure. The lower resolution of the former map is most probably due to the smaller number of sub-volumes but could also be caused by a higher flexibility of the protein. The model, arrived at through extensive comparison of many fits from multiple density fitting methods, placed the fusion loops at the top of the structure, facing away from the membrane. The here-presented EM map reveals densities in the center of the map (Fig. 4A), not seen previously, and the improved resolution now allows unambiguous fitting of DII to DIV. The connectivity of the domains therefore places DI pointing to the membrane as proposed for VSV-G (*12*). The previous model based on the wt gB protein likely consists of a large subset of conformational states. With a similar approach using cryo-ET on gB vesicles, Fontana *et al*. (*8*) obtained a map with a resolution of ~50 Å. The resolution of this map would have allowed both positions for DI to be possible, but combination of the inconclusive EM density with biochemical data, using monoclonal antibodies, led the authors to position DI pointing to the membrane reinforcing a previous, hypothetical model devised by fluorescent protein insertions (*34*). A recent cryo-EM map from SVA of human cytomegalovirus (HCMV) particles allowed determination of a prefusion conformation of HCMV gB at ~21-Å resolution (*10*). In contrast to our previous density map for HSV-1 gB showing a three-pointed crest at ~23-Å resolution, the HCMV gB map features a single central density at the top of the structure. This central density precludes fitting of DI at the top as this would lead to clashes within the trimer, and therefore, DI was fitted in the leg density pointing to the membrane. Our sub-nanometer–resolution map presented here now permits the unambiguous fit of all HSV-1 gB ectodomains, including DI pointing to the membrane (Figs. 5 and 6, and table S1), in agreement with the interpretation suggested by these studies (*8*, *10*).

The resolved density at the center of the map allowed placing of the complete DIII helix (Fig. 1A). However, fitting of the N-terminal part of the central helix of DIII (amino acids 515 to 545) located around the predicted bending point is challenging. The resolved density in the center of the EM map would accommodate the whole, unbroken helix, and our MD-based flexible fitting approach (*30*) suggests a kink near the predicted bending position at the N-terminal end of the helix, which would agree with a model for HCMV gB (*10*) but would be different from the hinge present in VSV-G and the strong local bending predicted by our MD simulation (Fig. 2). The central helix of DIII in gB of Herpesviridae contains a 9–amino acid insertion (Fig. 1B), which makes this helix substantially longer compared to the corresponding helix in VSV-G, also in the prefusion conformation, which would account for part of the density in the upper region of the map. Nevertheless, fitting the N-terminal region of this helix suffers from several issues: the low resolution of the EM map in this area, the 15–amino acid (476 to 491) linker region that is unresolved in the crystal structure, and the fact that this region likely undergoes an extensive local conformational change. It therefore remains to be determined to what extent the N-terminal part of this helix is bent.

DV, which is intrinsically less structured than DI to DIV, required substantial rearrangement, but the assignment is supported by connectivity to the membrane. Because of the overall quality of the fit, we assume that most of conformational changes between the pre- and postfusion form occur, as described for VSV-G, at interdomain connections rather than intradomain changes (*35*, *36*). The domain arrangement resulting from our domain fitting of gB resembles that of VSV-G in its prefusion conformation (Fig. 5) (*12*), although in gB, DIV is cyclically swapped within the trimer, making contacts with neighboring protomer subunits, while for the CD of VSV-G, such domain swapping does not occur (Fig. 5, top views). In VSV-G, comparing the pre- and the postfusion conformation, the FD and the C-terminal segment, which makes the connection to the transmembrane domain, flip around the CD, illustrating the relative domain rearrangement that occur during the fusion process (Fig. 5) (*35*). The domain rearrangement between the two conformations in gB follows a similar pattern. Here, DI, DII, and DV flip around the central part of the protein including the C-terminal region of DIII and DIV. In VSV-G, the CD stays essentially unchanged, and our model also predicts only minor changes for DIV and the C-terminal region of DIII during this transition in gB. In context of the gB trimer, the N-terminal regions DI and DII twist around the central axis of the trimer, which is not seen in VSV-G where there is hardly any lateral movement of the FD (Fig. 5, top views). Nevertheless, the general domain architecture of the ectodomains in the two conformations shows notable similarities that further corroborate the previously described homology (*37*) of these two proteins and the suggestion that these proteins evolved from a common ancestor. Other than VSV-G that fuses the viral membrane with the endosome membrane of the host only, gB can also fuse the HSV-1 envelope with the plasma membrane. The fact that both proteins accomplish their membrane fusion function in different contexts might explain the different triggering requirements (*35*) with VSV-G being activated by the low pH of the endosome, while HSV-1 gB fusion by low pH is not sufficient. For gB fusion, triggering seems more complex and regulated and is only possible as part of a four-protein machinery together with gD and gH/gL (*38*). The essential features of the conformational change—once triggered—have remained very similar.

The structural features we identified and exploited to lock gB are conserved in class III fusion proteins of other relevant pathogens, opening a path to stabilize their prefusion conformation and suggesting common structural rearrangements during fusion. Antibodies or antiviral drugs impairing this general rearrangement therefore have the capacity to achieve clinically relevant protection. Our results provide the first step for the rational design of prefusion epitope-focused, next-generation vaccines against important human pathogenic herpesviruses.

## MATERIALS AND METHODS

### Sequence alignment

Clustal Omega (*39*) was used to calculate the sequence alignment of central helices of glycoprotein G (DII) of animal Rhabdoviridae and gB (DIII) of human Herpesviridae with genus or subfamily representatives of the novirhabdoviruses [viral hemorrhagic septicemia virus (VHSV)], the lyssaviruses [rabies virus (RABV)], the ephemeroviruses [bovine ephemeral fever virus (BEFV)], the unclassified sigma virus of *Drosophila* (SIGV), and the vesiculoviruses [vesicular stomatitis indiana virus (VSV)], α-herpesviruses (human HSV-1 and VZV), γ-herpesviruses [EBV and Kaposi’s sarcoma-associated herpesvirus (HHV8)], and β-herpesviruses [human herpesvirus 6 (HHV6), HHV7, and HCMV]. Clustal Omega (*39*) was also used for the residue background coloring, which corresponds to conserved hydrophobic (blue), positively charged (red), negatively charged (magenta), polar (green), cysteines (pink), glycines (orange), prolines (yellow), aromatic (cyan), and unconserved (white) residues. Residue conservation was calculated using www.compbio.dundee.ac.uk/aacon/ (*40*).

### Expression plasmid construction

The sequence for a 5xGS linker was added to the C terminus of the gB gene, followed by a 6xHis tag in the pEP98 plasmid. The gene for VSV-G (strain San Juan) was amplified by polymerase chain reaction and inserted into the modified pEP98 vector, replacing gB. Single-point mutations were created using the Agilent QuikChange II Kit or NEB Q5 kit for site-directed mutagenesis.

### Vesicle preparation

Vesicles were prepared as described (*22*). In brief, BHK-21 cells were grown in GMEM (Glasgow’s Minimal Essential Medium) supplemented with 20 mM Hepes (pH 7.4), 2% (v/v) TPB (tryptose phosphate broth), and 2% (v/v) fetal bovine serum. At around 70% confluency, cells were transiently transfected. Cells were grown for an additional 48 hours with a media exchange to serum-free GMEM after 24 hours. Vesicles were harvested from the supernatant by differential centrifugation and resuspended in 20 mM Hepes (pH 8) and 150 mM NaCl.

### Expression tests

Vesicle preparations were tested in SDS–polyacrylamide gel electrophoresis (PAGE) followed by Coomassie staining or Western blotting with a rabbit anti-His6 antibody (Abcam) followed by anti-rabbit horseradish peroxidase (HRP) (Sigma-Aldrich Chemie GmbH). After supernatants were removed for vesicle preparations, cells were washed with cold phosphate-buffered saline (PBS) and detached using cell scrapers. Cells were pelleted by centrifugation (5 min, 4500*g*, 4°C), transferred into 1.5-ml tubes, and washed in cold PBS again before resuspension in radioimmunoprecipitation buffer, 100 μl per T175 flask [50 mM tris (pH 8), 1% NP-40, 0.1% SDS, 150 mM NaCl, 0.5% sodium deoxycholate, 5 mM EDTA, 1 mM phenylmethylsulfonyl fluoride]. Samples were shaken at 4°C for 30 min before being spun at 500*g* for 10 min. Supernatants were mixed in SDS sample buffer and run in parallel with vesicle samples in SDS-PAGE. For loading control, Western blots were re-probed using a mouse anti–glyceraldehyde-3-phosphate dehydrogenase antibody (Sigma-Aldrich Chemie GmbH), followed by anti-mouse HRP (Sigma-Aldrich Chemie GmbH).

### Transient transfection–based cell-cell fusion assay

Fusion activity of the different HSV-1 gB constructs was determined after transient transfection of RK13 cells as described recently (*41*). Briefly, cells were transfected with 200 ng each of the expression plasmids for enhanced green fluorescent protein (EGFP) (pEGFP-N1; Clontech), nectin-1, and HSV-1 glycoproteins gD, gL, gH, and gB or mutant gB in 100 μl of Opti-MEM using 1 μl of Lipofectamine 2000. Twenty-four hours after transfection, the cells were fixed with 3% paraformaldehyde and analyzed using an Eclipse Ti-S fluorescence microscope and NIS-Elements Imaging Software (Nikon). Fusion activity was determined by multiplication of the number of syncytia by the mean syncytia area within 10 fields of view (5.5 mm^2^ each). Each experiment was repeated four times, and average percent values of positive control transfections as well as standard deviations were calculated.

### MD simulations

Starting structures were generated by taking the DIII helix (residues 500 to 573) from the HSV-1 (postfusion) gB (2GUM) (*6*) structure. The helix was centered in a simulation cell large enough that no edge was closer than 2.0 nm from the protein, and subsequently solvated with SPC/E (extended simple point charge model) water (*42*) and neutralizing monovalent ions to a concentration of 0.15 M. Each complete system was first energy minimized using 50,000 steps of steepest descent energy minimization. It was then equilibrated in the *NVT* (number of particles, volume, temperature) ensemble at 300 K to bring the system up to the simulation temperature. The output was continued into the *NPT* (number of particles, pressure, temperature) ensemble equilibration at the same temperature and 1 atm isotropic pressure to bring the pressure to that required for the production simulation. Last, the *NPT* output was used as the starting structure and velocity distribution for the production MD simulations. MD simulation parameters: Leap-frog integrator was used to model the equations of motion, with a 2-fs time step. LINCS (linear constraint solver) was used to constrain all bonds involving hydrogen. Nonbonded van der Waals interactions were cut off using the Verlet scheme with a 1.0-nm cutoff, while electrostatics were modeled using the Particle Mesh Ewald (*43*) method with an order of 4 and 0.16 Fourier spacing. Temperature was coupled to an external velocity-rescale thermostat at 300 K, protein and nonprotein components being coupled separately, with a 0.1-ps time constant. For NPT equilibration and MD, pressure was isotropically set at 1 atm by coupling to a Parrinello-Rahman barostat with a 2.0-ps time constant. Equilibration simulations were run for 100 ps with position restraints applied to protein backbone atoms. The production MD simulations were run for 100 ns with no position restraints on the protein. All simulations were performed using Gromacs 5.1.2 (*44*). Local helix bending angles were determined using HELANAL (*45*), implemented within MDAnalysis (*46*, *47*) using a sliding window of four contiguous α carbons to compute local helix axes and origins over the length of the entire helix, one α carbon at a time. Bending angles are the angles between successive local helix axes. Local bending angle plots were generated with Matplotlib (*48*).

### Cryo-EM to determine vesicle size distributions

For grid preparation, 3.5 μl of vesicles were mixed with 10-nm gold fiducials on Quantifoil R2/1 grids and plunge-frozen in a propane/ethane mixture using a manual plunge freezer. Microscopy was performed using a Tecnai F30 “Polara” microscope (FEI Thermo Fisher Scientific) at 300 kV equipped with a Quantum 964 post-column energy filter (Gatan) operated in zero-loss imaging mode. Images were recorded on a 4k × 4k K2 Summit electron detector with a calibrated pixel size of 0.14 nm at the specimen level. Transmission images were recorded using SerialEM (*49*) at a −3-μm defocus. Vesicle diameters were measured in 3dmod.

### Tomogram acquisition

Tomograms were collected in 3° increments either bidirectionally on Tecnai F30 “Polara” or using a dose-symmetric scheme (*50*) on Titan Krios (FEI/Thermo Fisher Scientific) at 300 kV both with a 70-μm C2 aperture and post-column Quantum energy filter operated in zero-loss mode using an energy slit of 20 eV and K2 Summit direct electron detector in counting mode (Gatan). SerialEM (*49*, *51*) was used for automated data collection. Please see table S2 for data acquisition parameters.

### Image processing

Frames were aligned using Unblur (*52*) or SerialEM plugin (*51*). Defocus estimation was performed using IMOD ctfplotter (*53*), phase flipping was performed using NovaCTF (*54*) or ctfphaseflip, and dose weighting was performed using IMOD mtffilter. Tomograms were aligned using IMOD etomo (*53*) and subsequently reconstructed using NovaCTF, with the exception of eight tomograms that were used etomo’s local alignment. Two sets of tomograms were used with all available tilts (all-tilt tomograms) or using tilt series that had tilts collected above 20° and below −20° replaced by images filled with the tilt series mean intensity (low-tilt tomograms). Tomograms were binned two, four, or six times using IMOD binvol and filtered for visual inspection and manual picking in 3dmod (IMOD) using bfilter (*55*).

### Sub-volume averaging

Tomogram reconstruction was performed using IMOD (*53*) and NovaCTF (*54*). PEET (*55*, *56*) was used for SVA together with a set of bespoke scripts based on TEMPy (*57*) for particle coordinate manipulation. Two independent reference volumes were generated by aligning and symmetrizing particles from a small subset of tomograms binned six times and used for subsequent alignment of all particles within each half-dataset. All-tilt tomograms were replaced by low-tilt tomograms after convergence of two times binned data. Only translations were allowed for alignment of low-tilt tomograms due to a larger missing wedge. Alignment was initially focused on the whole protein including the membrane bilayer and then restricted to the protein ectodomain. Angles were refined at this stage using all-tilt tomograms, followed by further low-tilt translation refinement. Particle models were manually cleaned using UCSF (University of California, San Francisco) Chimera (*29*), and corresponding pairs of particles and orientations were removed from PEET models and initial motive lists, followed by a final alignment using unbinned tomograms. An average volume containing the protein CTD was obtained by expanding the alignment box and mask and allowing translation refinement. FSC (Fourier shell correlation) was calculated using bresolve (Bsoft) between the half-dataset averages masked with a binary mask using bmask (Bsoft) and a soft-edge mask using clip (IMOD) to avoid mask correlation. Because of a slight misalignment between the half-dataset averages, one half-map was aligned to the other in Chimera before FSC calculation. The 0.143 FSC cutoff was used to determine the ectodomain (9.0 Å) and the whole protein (10.1 Å) resolution. The final average containing both half-datasets (46,067 particles – post C3 symmarization) was generated by merging particles after manual cleaning and aligning positions to the sum of the half-dataset averages. The experimental *B* factor was estimated using embfactor (*58*) to ~400 Å^2^; a more conservative value of −300 Å^2^ was applied to combined average using bfilter (Bsoft).

### Model building

Rigid-body fitting of individual domains of the postfusion gB crystal structure (5V2S) (*14*) was performed using Chimera. The fit for DIV was used as a starting and reference point for symmetrization of protomer models. Fitting of domains was refined using Namdinator (*30*) with a sharpened SVA map and default parameters (20,000 steps). Visualization and figure generation were performed in Chimera or ChimeraX (*59*). Cross-correlations for each domain were calculated using the “Fit in Map” command in UCSF Chimera (*29*) for both: models generated by rigid-body fitting and MD flexible fitting (*29*), with a map simulated from the relevant atoms using a nominal resolution of 10 Å. The SCCC (segment-based cross correlation coefficient) was calculated using TEMPy (*57*), as implemented in the CCP-EM (Collaborative Computational Project for Electron cryo-Microscopy) software suite (*60*).

### Expression and crystallization of recombinant HSV-1 gB H516P ectodomains

The synthetic gene, codon-optimized for protein expression in insect cells, and coding for HSV-1 gB H516P residues 31 to 730 was cloned into the pT350 vector (*61*) between the vector-encoded Bip signal peptide that drives protein secretion and a double strep tag. The gB ectodomains were produced in S2 Drosophila cells using standard methods (*62*). The protein was purified on Strep-Tactin affinity resin and by size exclusion chromatography using Superdex 200 16/60 column and 10 mM tris (pH 8) and 50 mM NaCl as running buffer. The protein was concentrated to 6.4 mg/ml and crystallized in 0.1 M tris (pH 8), 18% ethanol at the Institut Pasteur core facility for crystallization (*63*). They were flash-frozen in liquid nitrogen in cryosolution containing 0.1 M tris (pH 8), 20% ethanol.

### X-ray data collection and structure determination of HSV-1 gB H516P ectodomain

The diffraction data were collected at the SOLEIL synchrotron source Proxima 2 beamline and processed with X-ray detector software (XDS) (*64*). The crystals belong to *P*321 space group, with cell dimensions *a* = 118 Å, *b* = 118 Å, *c =* 162 Å, α = β = 90°, and γ = 120°. The data were cut off at 6.4 Å [with cross correlation (CC)(1/2) value of 0.601 for the high-resolution shell] and used for molecular replacement done with Phaser (*65*) and the HSV1 gB structure (*6*) [Protein Data Bank (PDB) 2GUM] as the search model. One round of refinement was done using BUSTER (*66*).

## Supplementary Material

abc1726_Movie_S1.mov

abc1726_Movie_S2.mov

abc1726_SM.pdf

## SUPPLEMENTARY MATERIALS

Supplementary material for this article is available at http://advances.sciencemag.org/cgi/content/full/6/39/eabc1726/DC1

## Appendix

View/request a protocol for this paper from *Bio-protocol*.
