# Supplementary material for: The prefusion structure of herpes simplex virus glycoprotein B
Source: Sci Adv. 2020 Sep 25;6(39):eabc1726. doi: 10.1126/sciadv.abc1726 (PMC7518877; doi:10.1126/sciadv.abc1726)
Supplement: abc1726_SM.pdf [file abc1726_SM.pdf]

Science Advances
AAAS

advances.sciencemag.org/cgi/content/full/6/39/eabc1726/DC1

Supplementary Materials for

The prefusion structure of herpes simplex virus glycoprotein B

B. Vollmer, V. Pražák, D. Vasishtan, E. E. Jefferys, A. Hernandez-Duran, M. Vallbracht, B.G. Klupp, T. C. Mettenleiter, M. Backovic, F. A. Rey, M. Topf, K. Grünewald*

*Corresponding author. Email: kay.gruenewald@cssb-hamburg.de

Published 25 September 2020, *Sci. Adv.* **6**, eabc1726 (2020)
DOI: 10.1126/sciadv.abc1726

The PDF file includes:

Figs. S1 to S5
Tables S1 and S2
Legend for movies S1 and S2

Other Supplementary Material for this manuscript includes the following:

(available at advances.sciencemag.org/cgi/content/full/6/39/eabc1726/DC1)

Movies S1 and S2

Figure S1

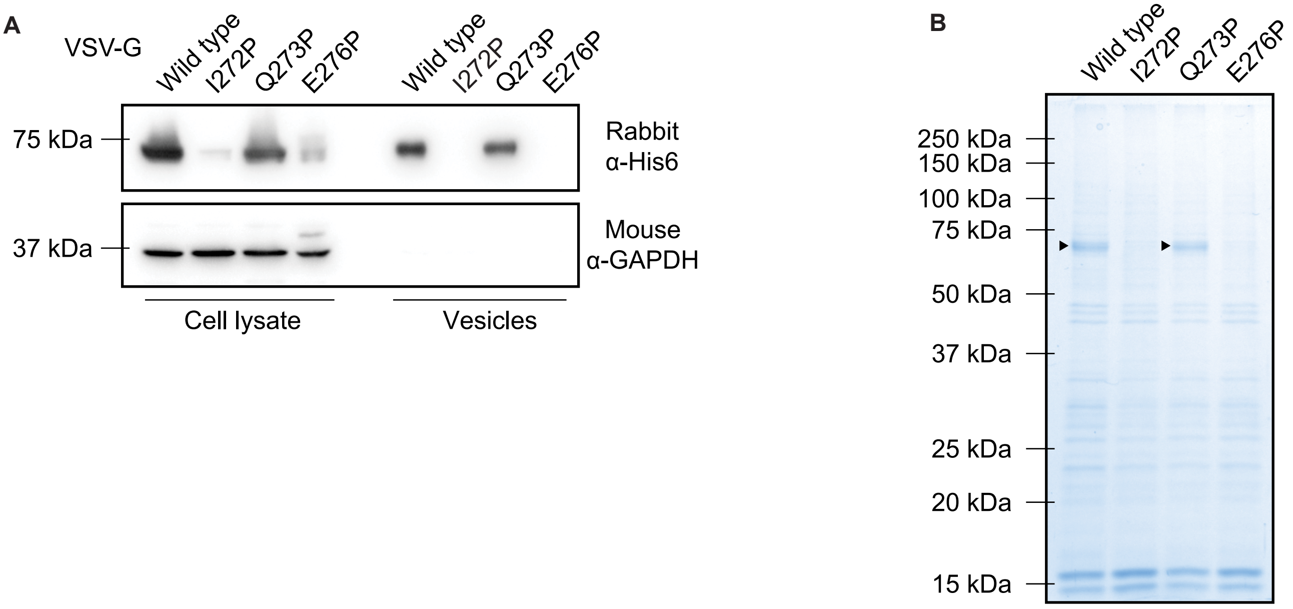

fig. S1 - Stabilizing the VSV-G CD helix hinge

(A) Western blot analysis of VSV-G WT and single point mutant expression from cell lysate and formation of purified extracellular vesicles.

(B) SDS-PAGE and Coomassie stain of purified extracellular vesicles, produced using VSV-G WT and single point mutants.

Figure S2

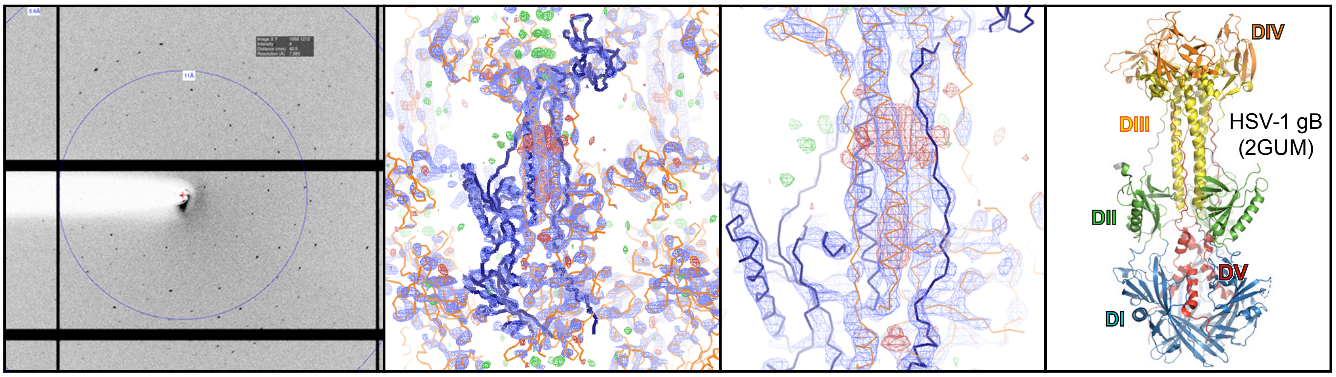

fig. S2 - Crystallization of gB H516P

X-ray diffraction pattern of gB H516P ectodomain crystal. Post-fusion X-ray structure (PDB: 2GUM) *(6)* was fitted in the acquired ~6 Å density map which is shown as ribbon model on the right, using the same color scheme as in Fig. 1A for comparison.

Figure S3

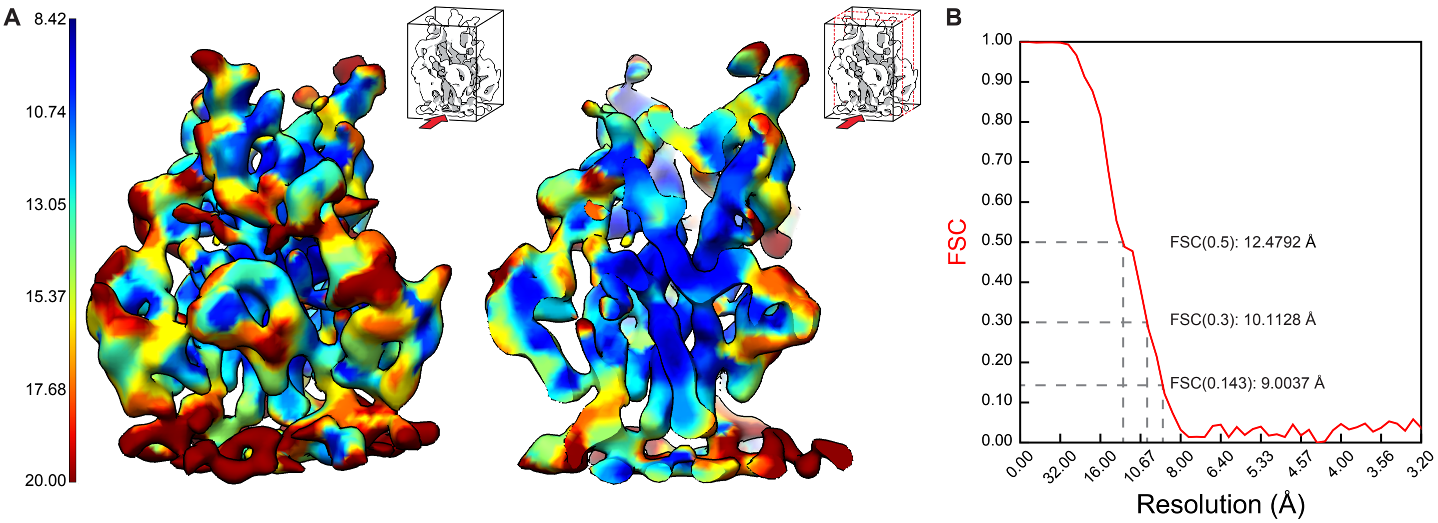

fig. S3 - Resolution determination for SVA of ectodomain gB H516P

(A) Local resolution map of the gB H516P ectodomain SVA structure.

(B) Resolution estimate using Fourier shell correlation of two half data set averages (46,067 particles total). Marked are resolutions at 0.5, 0.3 and 0.143 FSC.

Figure S4

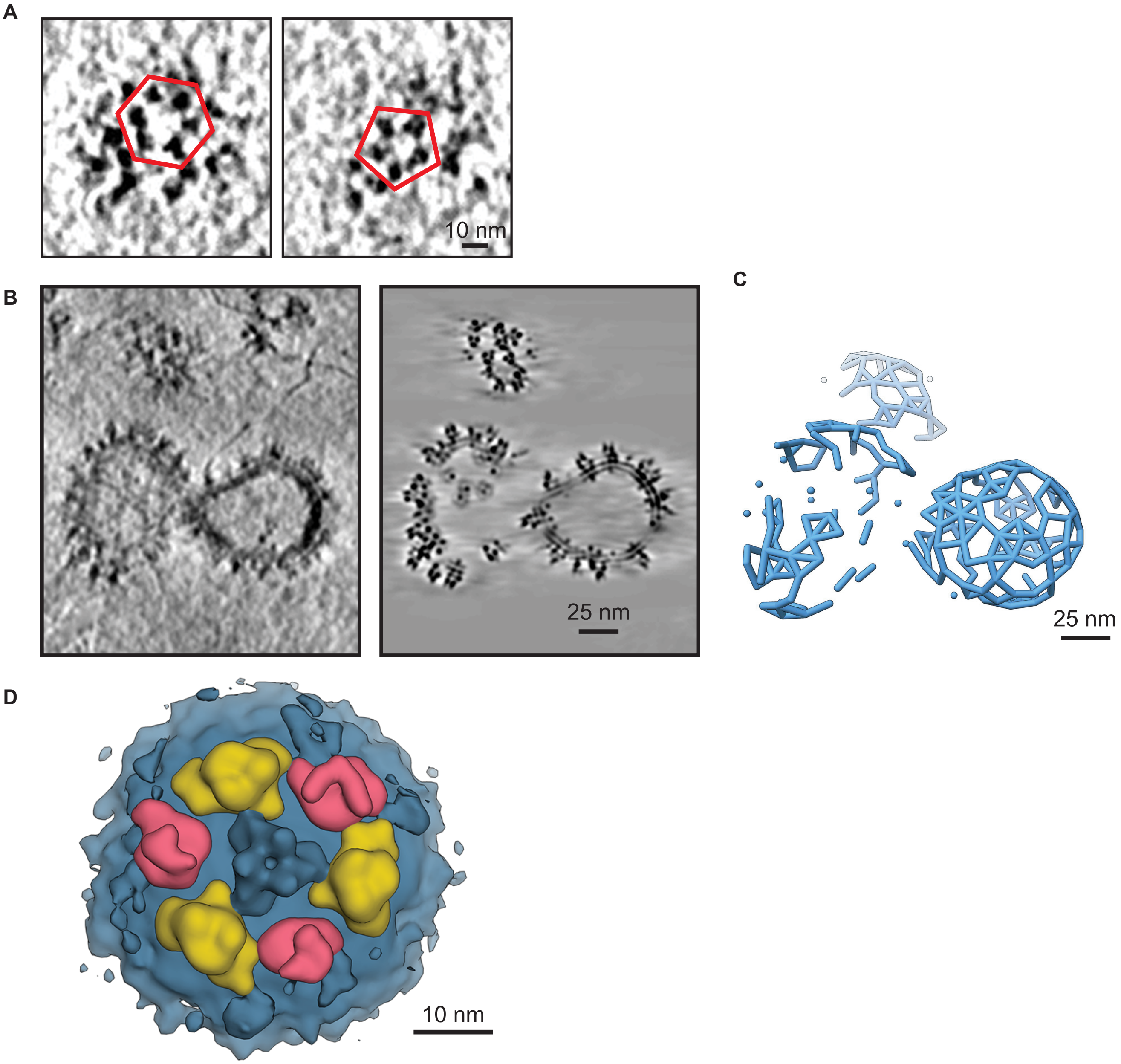

fig. S4 - Pre-fusion gB forms flexible homotypic interactions

(A) Tomogram slices indicating the presence of different arrangement of gB trimers on the surface of vesicles.

(B) A slice through tomogram (left) and a volume generated by backplotting SVA map into positions refined by SVA (right). Both volumes were filtered with the same parameters and have equivalent missing wedges.

(C) 3D model indicating the connectivity between gB trimers. Positions of particles (blue spheres) in tomogram shown in (B) shorter than 14 nm were linked by sticks.

(D) Top view of a SVA volume generated by aligning particles without using a mask. The volume was segmented in Chimera to indicate the position of 1° (yellow) and 2° neighbors.

Figure S5

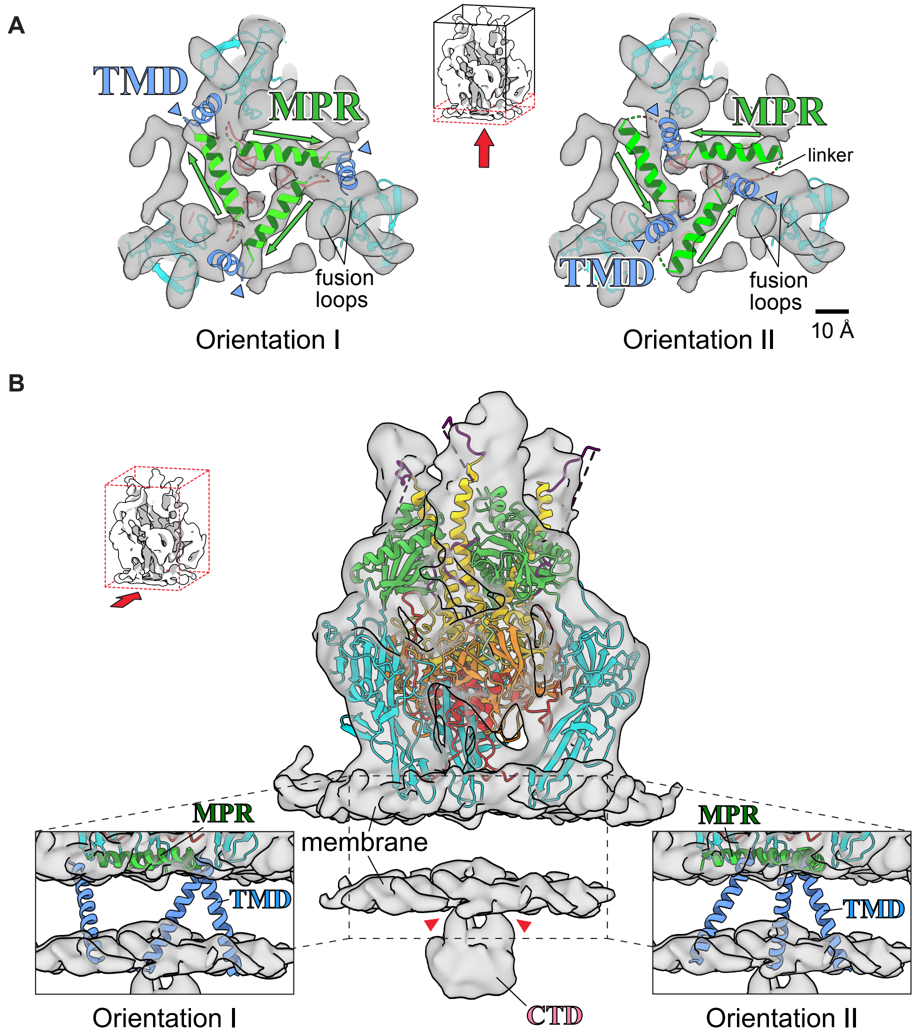

fig. S5 - SVA and fitting of full length gB H516P

(A) Bottom slice seen from below showing two possible orientations for the amphipathic helices of the MPR with hydrophobic residue positions shown in light green. Connection points to the TMD in blue are marked by blue arrowheads with a distance of 2.5 nm in Orientation I and 0.8 nm in Orientation II from the central 3-fold symmetry axis. Position of fusion loops of one protomer are indicated, respectively. Bar: 10Å.

(B) Front view of the full map including both membrane leaflets and cytoplasmic density, most probably representing the CTD. The connecting points of the CTD to the membrane are marked by red arrowheads. Due to the absence of density between the membrane leaflets, TMDs are shown in the two orientations implied by the MPR fit in the ectodomain (cf. panel C) and the CTD is not fitted.

Table S1 - Domain fit quality

**Rigid domain fit**							
Domain	aa range	CC chain A	CC chain B	CC chain C	**mean CC**	SD	
DI	154-363	0.607	0.608	0.612	**0.609**	0.003	
DI*	154-363	0.318	0.324	0.365	**0.336**	0.026	
DII	142-153,364-460	0.540	0.547	0.553	**0.547**	0.007	
DIII	501-571	0.494	0.493	0.493	**0.493**	0.001	
DIV	104-116,573-660	0.712	0.702	0.710	**0.708**	0.005	
DV	661-722	0.376	0.312	0.381	**0.357**	0.039	
**MD flexible fitting**							
Domain	aa range	CC chain A	CC chain B	CC chain C	**mean CC**	SD	**SCCC**
DI	154-363	0.684	0.696	0.705	**0.695**	0.011	**0.748**
DII	142-153,364-460	0.595	0.604	0.607	**0.602**	0.006	**0.750**
DIII	501-571	0.553	0.580	0.592	**0.575**	0.020	**0.716**
DIV	104-116,573-660	0.747	0.762	0.771	**0.760**	0.012	**0.705**
DV	661-722	0.400	0.376	0.411	**0.396**	0.018	**0.431**

Cross-correlations (CC) for each domain in each protomer (chain) were calculated for using UCSF Chimera 'Fit in Map', with a map simulated from the relevant atoms using a nominal resolution of 10 Å. Mean CC and standard deviation (SD) are given for each domain. SCCC was calculated using TEMPy implemented in the CCP-EM software suite. *DI fit in upper density.

Table S2 - Cryo-EM data collection and statistics

	Krios data	Polara data
**Data collection and processing**		
Voltage (kV)	300	300
Electron exposure (e–/Å2)	3.5	2.3 / 5.5*
Total dose	143.5	94.3 / 225.5
Defocus range (μm)	2.3-4.9	1.6-3.5
Pixel size (Å)	1.6 (0.9)**	1.35
Symmetry imposed	C3	C3
Acquisition scheme	dose-symmetric	bi-directional
Tilt increment	3°	3°
Tilt range	0° to +/- 60°	20° to -58° & 23° to 59°
Tomograms (no.)	46	53
Picked particles (no.)	37,897	18,279
Map resolution (Å)	9.0 Å	
FSC threshold	0.143	

*8 tomograms were collected with 5.5 e-Å-2 per tilt. **Krios data was collected at 0.9 Å pixel size at super-resolution mode, and Fourier cropped to 1.8 Å. Pixel size was then set to 1.6 Å after size comparison with averages from Polara data.

Movie S1

Plot of the local bending angles of domain III residues during molecular dynamic simulation. Residues are marked in one-letter code and one plot per ns is shown of a 100 ns long simulation.

Movie S2

The full length cryoET density map of gB, including the two leaflets of the membrane as well as the CTD is shown and in relation to the ectodomain-focused map. Horizontal slices through the density as depicted in Fig. 4A followed by sequential rigid body fitting of the individual domains. A surface rendering of the created model shows representative positions for the surface exposed N-linked glycosylations on the trimer followed by the domain architecture of an individual protomer as depicted in Fig. 6.
